# Symptom prevalence in gastrointestinal cancer: a secondary analysis of IPOS data

**DOI:** 10.1007/s00520-025-09919-3

**Published:** 2025-09-18

**Authors:** Elaine G. Boland, Assem Khamis, Khek Tjian Tay, Kathyrn Chater, Paul Taylor, Alison Landon, Joy Ross, Fliss E. M. Murtagh

**Affiliations:** 1 Hull University Teaching Hospitals NHS Trust, Hull, UK; 2 Wolfson Palliative Care Research Centre, Hull York Medical School, University of Hull, Hull, UK; 3 York and Scarborough Teaching Hospitals NHS Foundation Trust, York, UK; 4 St Luke’s Hospice, Centre for Health and Related Research, University of Sheffield, Sheffield, UK; 5 St Christopher’s Hospice, Sydenham, London UK

**Keywords:** Symptom prevalence, IPOS, Gastrointestinal cancer, Specialist palliative care, Community settings

## Abstract

**Purpose:**

Patients with gastro-intestinal (GI) cancer have a high symptom burden; however, comparative data to other cancers is lacking. The aim is to determine symptom prevalence for people with GI cancer receiving specialist palliative care in the community.

**Method:**

Secondary analysis of anonymised routinely collected symptom registry data from those receiving community-based specialist palliative care, between 2020 and 2023.

**Results:**

One thousand seven hundred thirty-three patients with GI cancer received 2,332 episodes of specialist palliative care. Common symptoms were: - pain (77% prevalence in GI cancer vs 73% other cancers); with 49% reporting being moderately/severely/overwhelmingly affected in GI vs 46% in other cancers. - nausea (34% in GI cancer vs 25% other cancers); with 16% moderately/severely/overwhelmingly affected in GI vs 11% in other cancers. - vomiting (17% in GI cancer vs 11% other cancers); with 8% moderately/severely/overwhelmingly affected in GI vs 5% in other cancers. - poor appetite (77% in GI cancer vs 68% other cancers); with 56% moderately/severely/overwhelmingly affected in GI vs 46% in other cancers. poor mobility (79% in GI cancer vs 84% other cancer); with 53% moderately/severely/overwhelmingly affected in GI vs 62% in other cancers.

**Conclusion:**

This novel study provides comparative evidence about the nature of the high symptom burden for those with GI cancer and shows that poor appetite, nausea, vomiting, and worse mobility are more prevalent compared to other cancers, while pain and weakness are of similar prevalence. The underlying reasons may relate to illness trajectory, referral timing, or other causes and need further exploration.

**Supplementary Information:**

The online version contains supplementary material available at 10.1007/s00520-025-09919-3.

## Introduction

Gastrointestinal (GI) cancers are a group of cancers affecting the digestive organs. According to the International Statistical Classification of Diseases and Related Health Problems 10th Revision (ICD-10), malignant neoplasms of digestive organs (codes C15–C26) include oesophagus, stomach, small intestines, colon, recto-sigmoid, rectum, anus and anal canal, liver, bile ducts, gallbladder and pancreas [1]. GI cancers represent 26% of global cancer incidence and 35% of cancer mortality [2]. In the UK, GI cancers contribute to 21% of cancer incidence and 27.5% of cancer deaths each year [3]. Ten-year survival rates are reported between 5% and 53% across types of GI cancers [3].

Patients with GI cancers experience high symptom burden toward the last year of life. In a cohort study looking at 11,242 patients with GI cancers, more than 50% of patients experienced moderate-severe tiredness, poor well-being, poor appetite, breathlessness, pain, and drowsiness [4]. The findings were similar in a study within an East Asian population, with fatigue, reduced oral intake, and anorexia reported as the most prevalent symptoms in patients with GI cancers towards the end of life [5]. In both studies, a reduction in symptoms was reported for GI cancer patients receiving specialist palliative care, in both inpatient and outpatient settings [4, 5]. A recent meta-analysis reported that in digestive cancers, the pooled prevalence of anxiety symptoms was 20.4%, while that of depression symptoms was 30.2%, thus contributing a negative effect on their psychological well-being [6].

To help identify patients’ symptoms and other concerns with advanced illness, healthcare providers utilise patient-reported outcome measurement tools. The Integrated Palliative Care Outcome Scale (IPOS) is a valid and reliable outcome measurement tool for those with advanced illness [7, 8]. It enables healthcare providers to assess and monitor symptoms and other concerns, as well as measure the impact of interventions [8].

Studies have shown that patients with GI cancer have a high symptom burden; however, comparative data to other cancer diagnoses is lacking [4, 5, 9–11]. It is postulated that patients with GI cancer have a higher symptom burden compared to other cancer diagnoses. The aim of the study is to determine the prevalence of symptoms in people with GI cancer receiving specialist palliative care in a community setting and compare the prevalence of symptoms with other cancers.

## Methods

### Design and settings

Using an established Outcomes Registry, secondary data analysis of routinely collected, anonymised symptom data from community-based specialist palliative care was performed between April 2020 and March 2023 in three sites: [1] London, [2] York, and [3] Sheffield. This is a prototype outcome registry for palliative care set up to test the feasibility of collecting routine outcomes data from palliative care services in the UK. Fifteen palliative care teams were invited to participate, and we report from those community palliative care teams who could provide large volumes of data (over 500 episodes of care).

### Ethical approval

This study utilised secondary data that were already anonymised and collected as part of routine care. In line with recommendations by the UK National Data Guardian on Data Security, Consent and Opt-Outs, and in accordance with our formal ethical approval for this Outcomes Registry study (East of England—Cambridge Central Research Ethics Committee, REC reference 20/EE/0188, IRAS reference 269,821), patients were not individually consented but were offered the opportunity to opt-out from their data being used as part of this Registry, including through NHS opt-out. This was in accordance with the Declaration of Helsinki.

### Definitions

The term ‘episode of care’ defines the time from first assessment to discharge or death. One episode of care may comprise one or multiple phases of illness [12]. The palliative phase of illness is a standardised categorisation, according to urgency of care needs of the individual, family, and the suitability of care plan [13]. The Australia-modified Karnofsky Performance Status (AKPS) scale is an 11-point scale validated to measure the patient’s performance across the dimensions of activity, work and self-care, and was used to measure the performance status at the point of care; a higher score equates to a better level of function (100 represents full function, down to 10 representing being comatose) [14].

### Participant eligibility criteria

We included any patient 18 years old or older and receiving specialist palliative care services from a community team. We excluded any patient who had incomplete episodes of care, which either started before April 2020 or were continuing beyond March 2023.

### Data sources and collection

Routinely collected outcomes data was extracted from the electronic system by IT personnel in each site, according to the Registry protocol. NHS numbers were anonymised, and patients who opted out of research (NHS national Opt Out) were excluded. Datasets included patient characteristics (age, sex, and ethnicity), clinical data (primary diagnosis, reason for referral), and episode of care data (number of contacts/week per episodes, episode duration, episodes outcomes, phase of illness, and IPOS).

### Statistical methods

We used descriptive statistics to calculate frequencies and percentages for categorical variables and mean and standard deviation (SD) or median and interquartile range (IQR) for continuous variables. We used chi-square tests to compare categorical variables and the *t*-test (for normally distributed data) or Mann–Whitney test (not normally distributed data) to compare continuous variables across GI cancers versus all other cancers. Then, we included all significant variables into a multivariable logistic regression to investigate the association between the independent variables and the dependent variable (having GI cancers vs. all other cancers) and to eliminate multiple testing error. Statistical analyses were performed using Stata 17.

## Results

Data was obtained from three UK community palliative care teams participating in the Registry. The cohort consisted of 1733 patients with GI cancers, who received 2332 episodes of care (Table 1); the median episode duration was 32 days (IQR 11–78). Three thousand eight hundred ten patients with other cancers (seen by the same teams) received 5033 episodes of care; the median episode duration was 36 days (IQR 12–89). The number of phases per episode was a median of three (IQR 1–4) in both groups. The median age in both groups was 74 years (IQR 63–83).

**Table 1:** Demographics of patients in cancer receiving specialist palliative care in community settings

	Patients with GI cancers	Patients with other cancers	t/χ	*p* value
	*N* = 2332	*N* = 5033		
Patients	1733	3810		
Site			5.5536	0.063
Site 1	1537 (65.9)	3456 (68.7)		
Site 2	223 (9.6)	444 (8.8)		
Site 3	572 (24.5)	1133 (22.5)		
Age				
Median (IQR)	74 (63–83)	74 (63–83)		
Mean ± SD	72 ± 13	72 ± 14	0.1160	0.9077
Min–Max	25–101	18–102		
Sex			34.653	< 0.001*
Female	1036 (44.4)	2607 (51.8)		
Male	1296 (55.6)	2426 (48.2)		
Ethnicity	*N* = 2303	*N* = 4975	4.6404	0.200
White	1690 (73.4)	3725 (74.9)		
Black or Black British	226 (9.8)	414 (8.3)		
Asian/Asian British	103 (4.5)	211 (4.2)		
Mixed/multiple ethnic groups	284 (12.3)	625 (12.6)		
Reason for referral*	*N* = 2088	*N* = 4527		
Pain/symptom control	1503 (72.0)	3185 (70.4)	1.144	0.253
Emotional/psychological/spiritual support	327 (15.7)	697 (15.4)	0.109	0.913
Palliative care	137 (6.7)	274 (6.1)	0.201	0.840
Rehabilitation	114 (5.5)	319 (7.0)	0.588	0.557
Advance care planning	72 (3.4)	143 (3.2)	0.113	0.910
Care in last days of life	71 (3.4)	153 (3.4)	0.008	0.994
Others	< 10 (-)	< 20 (-)		
Unknown code	< 10 (-)	< 10 (-)		
Number of phases per episode	*N* = 2319	*N* = 5002		
Median (IQR)	2 (1–4)	2 (1–4)	0.175	0.861
Mean ± SD	3.1 ± 2.4	3.1 ± 2.4		
Min–Max	1–25	1–21		
Contacts frequency/week				
Median (IQR)	1.6 (0.8–3.5)	1.4 (0.7–3.3)	4.556	< 0.001*
Mean ± SD	2.9 ± 3.7	2.7 ± 3.5		
Min–Max	0.1–42	0.1–39		
Episode duration				
Median (IQR)	32 (11–78)	36 (12–89)	2.739	0.006*
Mean ± SD	60 ± 79	67 ± 87		
Min–Max	1–959	1–844		
Episode result	*N* = 2328	*N* = 5032	0.1375	0.711
Died	966 (41.5)	2065 (41.0)		
Discharged	1362 (58.5)	2967 (59.0)		
Place of death	*N* = 1108	*N* = 2302	9.1005	0.059
Home	785 (70.8)	1542 (67.0)		
Hospital	194 (17.5)	449 (19.5)		
Nursing/residential/care home	110 (9.9)	285 (12.4)		
Hospice	< 20 (-)	< 30 (-)		
Others	< 10 (-)	< 10 (-)		
AKPS	*N* = 1757	*N* = 3780	5.242	0.022*
≤ 30	326 (18.5)	802 (21.2)		
≥ 40	1431 (81.5)	2978 (78.8)		
Phase of illness at episode start	*N* = 2265	*N* = 4888	2.1271	0.712
Stable	477 (21.1)	1069 (21.9)		
Unstable	614 (27.1)	1288 (26.3)		
Deteriorating	1072 (47.3)	2320 (47.5)		
Dying	102 (4.5)	211 (4.3)		

^*^More than one option could apply

Over 70% of referrals were for pain and other symptom control. Regarding AKPS, 1431 (81.5%) patients with GI cancers vs. 2978 (78.8%) patients in other cancers had a score of ≥ 40. There was no significant difference (at the 5% level) between patients with GI cancer and patients with other cancers in relation to whether they were discharged or died at episode end (died 41.5% vs. 41.0% and discharged 58.5% vs. 59.9%; *p* = 0.711). Site 3 showed significantly higher cases of GI cancer compared to site 1 (OR 1.15 (1.02–1.30).

**Fig. 1:**
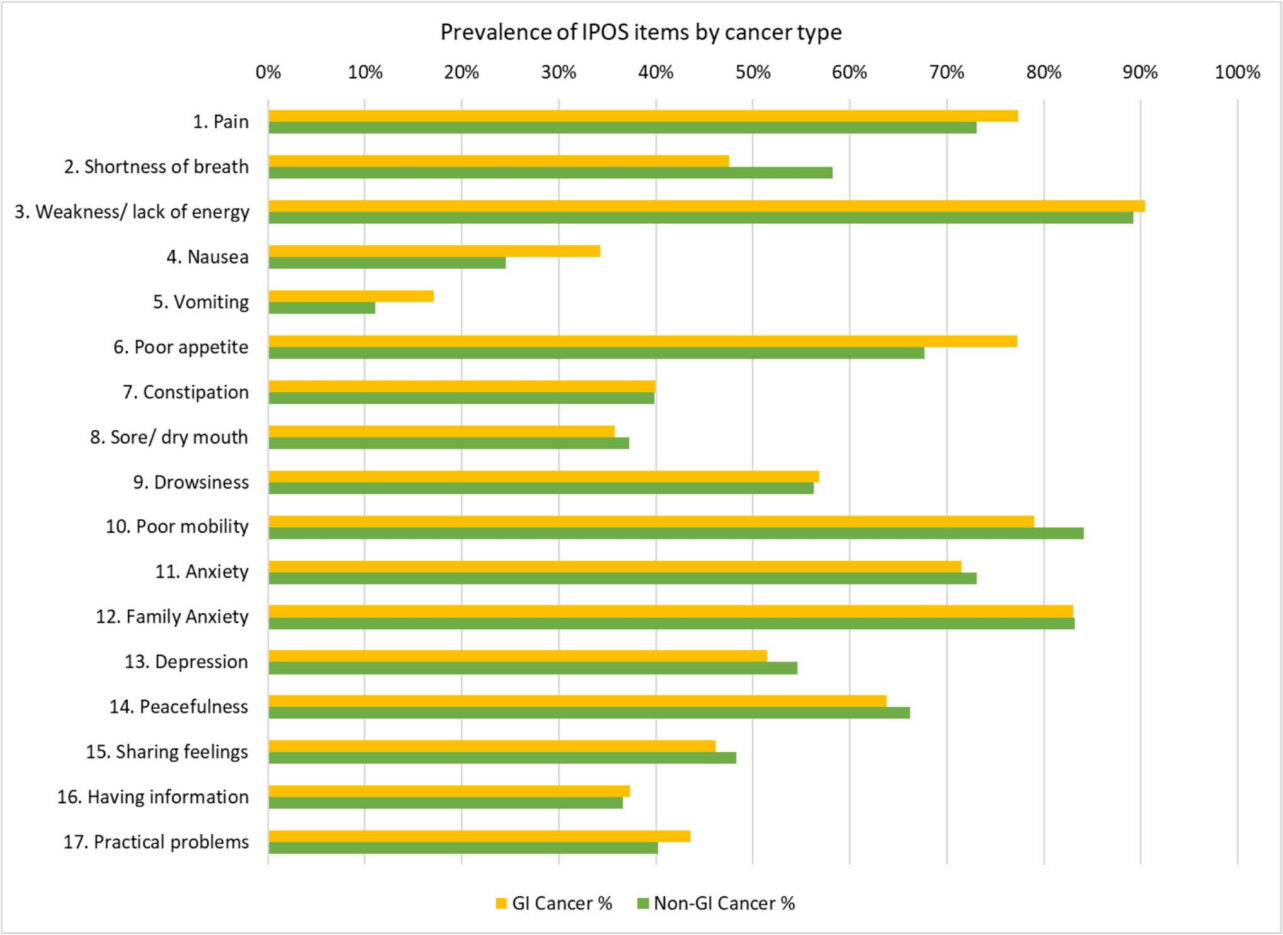
Prevalence of IPOS symptoms and concerns in patients with GI cancer and all other cancers

The most common symptoms reported (Fig. 1) were as follows:

- Pain 77% (95% CI 75–79) prevalence in GI cancer vs. 73% (95% CI 72–75) other cancers, with 49% (95% CI 47–52) reporting being moderately/severely/overwhelmingly affected in GI vs. 46% (95% CI 44–48) in other cancers
- Nausea 34% (95% CI 32–37) in GI cancer vs. 25% (95% CI 23–26) other cancers, with 16% (95% CI 14–18) moderately/severely/overwhelmingly affected in GI vs. 11% (95% CI 10–12) in other cancers
- Vomiting 17% (95% CI 15–19) in GI cancer vs. 11% (95% CI 10–12) other cancers, with 8% (95% CI 7–9) moderately/severely/overwhelmingly affected in GI vs. 5% (95% CI 4–5) in other cancers
- Poor appetite 77% (95% CI 75–79) in GI cancer vs. 68% (95% CI 66–69) other cancers, with 56% (95% CI 54–59) moderately/severely/overwhelmingly affected in GI vs. 46% (95% CI 45–48) in other cancers
- Weakness/lack of energy 90% (95% CI 89–92) prevalence in GI cancer vs. 89% (95% CI 88–90) other cancers, with 71–72% (95% CI 69–74) moderately/severely/overwhelmingly affected for both GI and other cancers
- Poor mobility 79% (95% CI 77–81) in GI cancer vs. 84% (95% CI 83–85) other cancer, with 53% (95% CI 50–55) moderately/severely/overwhelmingly affected in GI vs. 62% (95% CI 60–64) in other cancers

With regard to psychological issues, the prevalence of anxiety was 71% (95% CI 69–74) in GI cancer vs. 73% (95% CI 71–74) other cancers, with 44% moderate/severely affected in GI cancer (95% CI 41–47) vs. 46% (95% CI 44–48) in other cancers. On the other hand, the prevalence of depression was 51% (95% CI 48–55) with GI cancer vs. 55% (95% CI 52–57) with other cancers, with 25% (95% CI 22–27) moderate/severely affected in GI vs. 28% (95% CI 26–30) in other cancers.

Table 2 describes the demographics with regards to disease-specific groups, and the diseases have been coded using the ICD-10 (the International Classification of Diseases, 10th Revision) [1].

**Table 2:** Patient characteristics in disease specific groups

	Cancer of liver, intrahepatic bile ducts, gallbladder [C22-C24]*	Cancer of the digestive organs, including colon, rectum, stomach [C15–C21 & C26]*	Cancer of the pancreas [C25]*
	*N* = 274	*N* = 1678	*N* = 380
Age			
Median (IQR)	73 (63–82)	74 (63–83)	73 (62–81)
Mean ± SD	72 ± 13	71 ± 14	71 ± 12
Min–Max	32–96	25–101	31–97
Sex			
Female	112 (40.9)	724 (43.1)	200 (52.6)
Male	162 (59.1)	954 (56.9)	180 (47.4)

^*^We used the ICD-10 (the International Classification of Diseases, 10th Revision) for coding of the diseases

Detailed data on overall symptom prevalence stratified by cancer subtype are provided in Supplementary Table 1.

Table 3 shows the associations between patients’ characteristics and having GI cancers vs. all other types of cancer. Being male or being black or black British was associated with a higher risk of GI cancers. Patients with GI cancers had a significantly shorter episode of specialist palliative care compared to other cancers by—on average—4 days (GI cancers 32 days vs. other cancers 36 days; adjusted OR 0.99 (0.99–0.99), *z *value 2.27, *p* = 0.023).

**Table 3:** Association between patients’ characteristics and having GI cancer vs. all other types of cancer

Dependent variable: having GI cancer vs. all other cancer	Adjusted OR (95% CI)	*z* value	*p* value
Sex			
Female	1		
Male	1.34 (1.22–1.48)	5.82	< 0.001*
Ethnicity			
White	1		
Black or Black British	1.27 (1.07–1.52)	2.70	0.001*
Asian/Asian British	1.11 (0.86–1.41)	0.80	0.424
Mixed/multiple ethnic groups	0.97 (0.82–1.14)	0.38	0.704
Contacts frequency/week	1.01 (0.99–1.02)	1.26	0.206
Episode duration	0.99 (0.99–0.99)	2.27	0.023*
Site			
Site 1	1		
Site 2	1.17 (0.96–1.43)	1.57	0.116
Site 3	1.15 (1.02–1.30)	2.28	0.023*

Regarding dropouts and incomplete episodes, the average opt-out rate across sites was 6.3%, while the proportion of incomplete episodes was 6.2%.

## Discussion

This novel study reports the high symptom burden for those people with GI cancer who have been referred to community palliative care teams in three different locations in the UK and compares this symptom prevalence with other cancers. It is notable, but perhaps expected, that those people with GI cancers have a higher burden of certain symptoms, specifically gastro-intestinal symptoms such as poor appetite, nausea, and vomiting. We also report shorter episodes of community-based palliative care compared to other cancers, although it is not clear whether this may reflect differences in the trajectory of illness, later referral for care, or some other cause; similar proportions in both groups were discharged or died at episode end. Levels of pain and weakness were similar regardless of cancer type. We also demonstrated that sex (being male) and ethnicity (Black or Black British) are associated with GI cancer in this population receiving community-based specialist palliative care.

Most studies report a high prevalence of symptoms for people with all cancer types; a systematic review of symptom prevalence for people with incurable cancer showed that pain, lack of energy, fatigue, weakness, and appetite loss were the most frequent symptoms and occurred in > 50% [15, 16]. In addition to disease-related symptoms, treatment-related symptoms contribute to symptom burden for those with cancer [9]. A prospective study showed high treatment-related symptoms in upper GI cancer patients undergoing active cancer treatment [10]. Comparatively, more patients undergoing active cancer treatment reported moderate to severe symptoms compared to those with no active treatment (35.2% vs. 27%) [11].

In advanced gastrointestinal cancer, a study using the Edmonton Symptom Assessment System (a common symptom measure) showed moderate and/or severe symptom prevalence of fatigue (41.6%), pain (32.4%), drowsiness (28.4%), lack of appetite (25.5%), constipation (24.5%), anxiety (24.5%), and depression (21.6%), just prior to treatment [16]. Regarding ethnicity, prior evidence has shown a mixed picture. A national study in the UK showed that the incidence of pancreatic, colorectal, and oesophageal cancer was lower in the ‘non-white’ groups compared to those with white ethnicity, with a higher incidence of liver and gallbladder cancer among white groups, whereas those with a black ethnic background had a higher incidence of gastric cancer [17].

A longitudinal population analysis of access to hospital and community palliative care for patients with advanced cancer reported that the average duration of palliative care involvement was 6 weeks and the average number of contacts was two [18], while the duration of palliative care services in UK community and hospital settings before death identified the median duration of specialist palliative care for patients with cancer was 37 days [19]. In our study, the median episode duration was similar to this national study: 32 days in GI cancers vs. 36 days in other cancers. An international systematic review and meta-analysis involving 11,996,479 patients from 23 countries showed in contrast that the median duration from the start of palliative care to death was markedly less, at 18.9 days (IQR 0.1), (15 days for cancer), and this also depended on the setting (19 days for specialist palliative care unit, 20 days for community/home care, and 6 days for general hospital ward). If palliative care is provided earlier, at least 3–6 months prior to death, evidence shows that it has the most benefit to improve the patient’s quality of life and lessen the symptom burden [20–24]. 

A strength of this study is that data was collected prospectively using IPOS and in a ‘real-world’ clinical context. The large numbers were possible through the use of Outcomes Registry data; building the Outcomes Registry has only been possible through sustained research and implementation work, with extensive collaborative effort. Our study relies on data from three specific sites and community-based palliative care services, which may not be representative of patients receiving care in other settings. Another limitation of this study is that we do not know the reasons for differences observed, for instance, the cause of shorter episodes of care or how factors interact. However, our study design does not allow us to determine whether this reflects later referral to palliative care, differences in disease trajectory, or other unmeasured factors. A more detailed understanding of referral timing and its influence on symptom management would be valuable in future research.

## Conclusion

Uncertainties still arise about the best model of care that should be delivered to this cohort of patients with GI cancers and when integration with palliative care should occur. However, the high symptom burden—notably greater than with other cancers, and specifically in relation to gastrointestinal symptoms—indicates that early referral to specialist palliative care is important.

## Supplementary Information

Below is the link to the electronic supplementary material.

ESM 1 (DOCX 18.3 KB)

## Data Availability

No datasets were generated or analysed during the current study.
