# Supplementary material for: Symptom prevalence in gastrointestinal cancer: a secondary analysis of IPOS data
Source: Support Care Cancer. 2025 Sep 18;33(10):860. doi: 10.1007/s00520-025-09919-3 (PMC12443883; doi:10.1007/s00520-025-09919-3)
Supplement: Supplementary file 1 — (DOCX 18.3 KB) [file 520_2025_9919_MOESM1_ESM.docx]

**Title** Symptom prevalence in gastrointestinal cancer: a secondary analysis of IPOS data

Elaine. G. Boland MD, PhD, FRCP ^1,2^, Assem. Khamis MD, MPH ^2^, Khek Tjian Tay MBChB ^1^, Kathyrn Chater PhD, MSc, PGDip, BSc, RN ^3^, Paul Taylor MB ChB, PhD ^4^, Alison Landon MBChB ^5^, Joy Ross MBBS PhD FRCP ^2, 5^, Fliss E.M. Murtagh PhD, MD^2^

1. Hull University Teaching Hospitals NHS Trust, Hull, UK
2. Wolfson Palliative Care Research Centre, Hull York Medical School, University of Hull, Hull, UK 
3. York and Scarborough Teaching Hospitals NHS Foundation Trust, York, UK
4. St Luke's Hospice, Sheffield & Sheffield Centre for Health and Related Research, University of Sheffield, Sheffield, UK

5. St Christopher’s Hospice, Sydenham, London, UK

Corresponding author: Dr Elaine G Boland, Hull University Teaching Hospitals NHS Trust, Hull, UK
[Elaine.boland1@nhs.net](mailto:Elaine.boland1@nhs.net)
ORCID ID: 0000-0003-2571-5929

Supplementary table: Overall symptom prevalence in different cancer subtypes.

|  | Total patients with cancer of the digestive organs, including colon, rectum, stomach  [C15 – C21 & C26] | Total % of patients | 95% CI | Total patients with cancer of liver, intrahepatic bile ducts, gallbladder [C22-C24] | Total % of patients | 95% CI | Total patients with cancer of the pancreas [C25] | Total % of patients | 95% CI |
| --- | --- | --- | --- | --- | --- | --- | --- | --- | --- |
| Pain | 834 | 76% | 73-79% | 125 | 77% | 71-84% | 207 | 83% | 79-88% |
| Shortness of breath | 538 | 50% | 47-53% | 64 | 40% | 32-47% | 101 | 42% | 36-48% |
| Weakness/ lack of energy | 970 | 90% | 89-92% | 138 | 88% | 83-93% | 222 | 92% | 89-98% |
| Nausea | 355 | 33% | 30-36% | 58 | 37% | 29-44% | 95 | 39% | 33-45% |
| Vomiting | 189 | 17% | 15-20% | 25 | 16% | 10-21% | 40 | 17% | 12-21% |
| Poor appetite | 831 | 77% | 75-80% | 122 | 76% | 70-83% | 190 | 79% | 74-84% |
| Constipation | 418 | 39% | 36-42% | 57 | 37% | 30-45% | 103 | 44% | 38-51% |
| Sore/ dry mouth | 357 | 35% | 32-38% | 50 | 34% | 27-42% | 87 | 38% | 32-45% |
| Drowsiness | 574 | 56% | 53-59% | 79 | 54% | 46-62% | 144 | 62% | 55-68% |
| Poor mobility | 850 | 80% | 78-83% | 126 | 83% | 78-89% | 161 | 70% | 64-76% |
| Anxiety | 558 | 73% | 70-76% | 59 | 64% | 54-74% | 107 | 67% | 60-75% |
| Family Anxiety | 612 | 84% | 81-86% | 81 | 84% | 77-92% | 120 | 79% | 72-85% |
| Depression | 391 | 53% | 49-57% | 46 | 51% | 41-61% | 64 | 44% | 36-52% |
| Peacefulness | 449 | 66% | 62-70% | 47 | 56% | 45-67% | 77 | 57% | 49-66% |
| Sharing feelings | 330 | 47% | 43-50% | 41 | 48% | 37-58% | 58 | 43% | 35-51% |
| Having information | 261 | 38% | 35-42% | 29 | 34% | 24-44% | 44 | 35% | 26-43% |
| Practical problems | 319 | 46% | 42-50% | 28 | 33% | 23-43% | 46 | 37% | 28-45% |
